# Changes of liver hemodynamic and elastography parameters in patients with colorectal liver metastases receiving preoperative chemotherapy: “a note of caution”

**DOI:** 10.1186/s12957-017-1290-5

**Published:** 2017-12-16

**Authors:** Amalia Pelegrina, Josep Martí, Rosa Miquel, Joana Ferrer, Virginia Hernández-Gea, Alba Diaz, Cristina Nadal, Juan Carlos García-Valdecasas, Josep Fuster

**Affiliations:** 1 Liver Surgery and Transplant Unit, Department of Surgery, Institut de Malalties Digestives i Metabòliques Hospital Clinic Barcelona, IDIBAPS, University of Barcelona, Barcelona, Spain; 2 Barcelona Hemodynamics Laboratory, Liver Unit, IDIBAPS, CIBERehd, Hospital Clínic, University of Barcelona, Barcelona, Spain; 3 Network for Biomedical Research in Hepatic and Digestive Diseases (CIBERehd), Madrid, Spain; 4 Department of Pathology, Hospital Clinic Barcelona, IDIBAPS, University of Barcelona, Barcelona, Spain; 5 Institut de Malalties Hemato-Oncológiques Hospital Clinic Barcelona, IDIBAPS, University of Barcelona, Barcelona, Spain; 6 Institute of Liver Studies King’s College Hospital, Denmark Hill, SE5 9RS UK

**Keywords:** Colorectal cancer, Liver metastases, Hepatic hemodynamic, Hepatic veins pressure gradient, Oxaliplatin, Sinusoidal obstruction syndrome

## Abstract

**Background:**

New systemic chemotherapy agents have improved prognosis in patients with colorectal liver metastases (CLM), but some of them damage the liver parenchyma and ultimately increase postoperative morbidity and mortality after liver resection. The aims of our study were to determine the degree of hemodynamic and pathological liver injury in CLM patients receiving preoperative chemotherapy and to identify an association between these injuries and postoperative complications after liver resection.

**Methods:**

This is a prospective descriptive study of patients with CLM receiving preoperative chemotherapy before curative liver resection from November 2013 to June 2014. All patients had preoperative elastography and hepatic hemodynamic evaluation. We analyzed clinical preoperative data and postoperative outcomes after grouping the patients by chemotherapy type, development of sinusoidal obstructive syndrome (SOS), and development of major complications.

**Results:**

Eleven from the 20 patients included in the study received preoperative oxaliplatin-based chemotherapy (OBC). Nine patients had SOS at pathological analysis and five patients developed major complications. Patients receiving preoperative OBC had higher values of hepatic venous pressure gradient (HVPG) and developed more SOS and major complications. Patients developing SOS had higher values of HVPG and developed more major complications. Patients with major complications had higher values of HVPG, and patients with a HVPG of 5 mmHg or greater had more major complications than those under 5 mmHg (20 vs 80%, *p* = 0.005).

**Conclusions:**

OBC and SOS impair liver hemodynamics in CLM patients. An increase in major complications after liver resection in these patients develops at subclinical HVPG levels.

## Background

Colorectal carcinoma (CRC) is one of the most common cancers in the Western world, and approximately 50% of patients develop liver metastases [1]. The standard treatment for resectable colorectal liver metastases (CLM) is complete surgical resection, which offers a 5-year survival rate up to 58% [2]. In the last years, new systemic chemotherapy agents have improved the rates of complete resection and survival in patients with unresectable CLM at the time of diagnosis [3]. However, some of these agents damage the liver parenchyma and increase postoperative morbidity and mortality [4].

The main patterns of liver injury from systemic chemotherapeutic agents are chemotherapy-associated steatohepatitis and sinusoidal obstructive syndrome (SOS), which are associated with irinotecan and oxaliplatin respectively [4, 5]. Liver damage remains asymptomatic in the majority of cases, but severe oxaliplatin-induced SOS cases can evolve into non-cirrhotic portal hypertension (PHT) and ultimately complicate the safe application of subsequent therapies [4, 6]. Recently, several studies have found promising biochemical markers to diagnose the presence of sinusoidal obstruction, but these biomarkers could not quantify the degree and extent of hepatic injury nor diagnose the presence of clinical or subclinical PHT. Moreover, none of these studies measured the direct hemodynamic effects of chemotherapy-induced hepatic toxicity or quantified its related postoperative risks [7, 8].

Therefore, the aims of our study were to determine the hemodynamic impairment, liver stiffness, and pathological liver injury in patients with CLM who received preoperative chemotherapy and to identify a possible link between these injuries and the postoperative complications after liver resection.

## Methods

This is a prospective descriptive study of patients with CLM who received preoperative chemotherapy and underwent liver resection with curative aim between November 2013 and June 2014 at our Liver Surgery and Transplantation Unit. As per the study, all patients had hepatic elastography and hemodynamic measurements to assess for liver fibrosis and hepatic venous pressure gradient (HVPG) before undergoing liver resection. We excluded patients with any pre-existing liver condition (e.g., cirrhosis, chronic liver disease) and patients with previous liver resection. The hospital ethics committee approved this study, and all included patients gave their informed consent to participate in the study when surgery for CLM was proposed.

We obtained all demographical, clinical, and pathologic data from our prospective CLM database and patients’ charts. Preoperative chemotherapy (defined as received within the 6 months before CLM surgery) regimen was decided by the referring oncologist based on institutional protocols and routine practice. Patients underwent protocolized preoperative CLM staging (imaging and blood tests including tumor markers and liver function tests), postoperative care, and follow-up as previously described [9]. All patients were appointed to the Liver Surgery and Transplantation Clinic 1 month after discharge to record and grade the presence of complications during this postoperative period according to the Clavien-Dindo classification [10]. As per our Institution policy [9], CLM diagnosed before, during, or within 90 days of CRC resection were classified as synchronous and those CLM diagnosed at least 90 days after CRC resection were classified as metachronous. We defined major hepatectomy as the resection of three or more liver segments.

HVPG was obtained in all patients through a standard transjugular approach under local anesthesia. Briefly, we placed an 8-Fr venous catheter introducer in the right internal jugular vein with the Seldinger technique, measured wedge and free hepatic venous pressures, and calculated HVPG as the difference between them. We defined HVPG as normal when it was under 5 mmHg, as subclinical PHT when HVPG was between 6 and 9 mmHg, and as clinically significant PHT when HVPG was 10 mmHg or more regardless of the presence of clinical signs of PHT [11].

Elastography was obtained in all patients lying in the dorsal decubitus position with the right arm in maximal abduction. The measures were performed on the right lobe of the liver through intercostal spaces and values expressed in kilopascals (kPa) as previously described [12]. We defined normal elastography as stiffness under 7 kPa, presence of fibrosis when stiffness was between 7 and 14 kPa, and cirrhosis when stiffness was over 14 kPa.

Histological analysis was performed by a local experienced liver pathologist who was unaware of the treatment modality. Surgical specimens were processed and sampled as routinely according internal cut-up protocols. The number of tumors, size, distance to the transection margin, and capsular involvement were recorded, as well as the presence, distribution and approximate percentage of reticulated areas of congestion or hemorrhage, or parenchymal nodularity. At least one sample of non-tumoral liver more than 2 cm distant from the tumor was taken, with representation of the macroscopic lesions if present. The microscopic evaluation of the lesions on the background parenchyma was based on hematoxylin-eosin, Masson’s trichrome, and reticulin stains. SOS was diagnosed and graded according to previously published criteria [13]. Briefly, the diagnosis of SOS was made when sinusoidal dilatation, sinusoidal congestion, and hepatocellular atrophy coexisted. Other lesions such as endothelial rupture with erythrocyte extravasation at the Disse space, central vein obliteration, nodular regenerative hyperplasia, and perisinusoidal fibrosis were also recorded. Finally, the degree of steatosis was assessed if present according to previous published criteria [14].

The patients were classified into different groups according to whether they had or not received oxaliplatin-based chemotherapy (OBC) (thus, investigated and control groups respectively), according to the presence of SOS at the pathology exam, and whether they had major postoperative complications (defined as grade III or higher in the Clavien-Dindo classification during the 30 days after the CLM operation).

All quantitative variables were tested with the Shapiro-Wilk test to ascertain normal distributions. We described quantitative variables as mean (standard deviation) or as median (interquartile range) when distribution was not normal. Qualitative variables were expressed as number and % and compared with chi-square test. We compared groups with Student’s *t* test, Mann-Whitney U test or Fisher’s exact test as appropriate. A *p* value under 0.05 was considered statistically significant. All statistical calculations were performed with the “Statistical Package for the Social Sciences” version 22.0 for Windows (SPSS, Chicago, IL, USA).

## Results

### Patients’ characteristics

Twenty patients fulfilled the inclusion criteria during the study period. Demographic and preoperative data of the patients are shown on Tables 1 and 2. Eleven patients (55%) received preoperative OBC, with two of these patients receiving also biological agents (one panitumumab and another one bevacizumab). The median number of cycles of chemotherapy per patient was 6 (range 1–12). In three patients of the series, prothrombin time was under 80%, and only four patients had platelets under 150,000, although all patients had platelets over 100,000. The majority of patients had normal HVPG values, and no patients had significant PHT. Three patients (15%) had liver stiffness over 7 kPa, but no patients had stiffness values over 9 kPa.

**Table 1:** Patients characteristics (*n* = 20)

Sex (male:female)	13:7
Age (years) (mean ± SD)	63.35 ± 11.49
Body mass index (kg/m^2^) (median, range)	25 (22.9–29.5)
ASA grade (*n*, %)	
I–II	12 (60%)
III–IV	8 (40%)
Platelets (mean ± SD)	208,600 ± 73,650
Prothrombin activity percentage (mean ± SD)	94.47 ± 10.44
HVPG (mmHg) (mean ± SD)	3.80 ± 1.67
Elastography (kPa) (mean ± SD)	5.41 ± 1.67
No. of metastases (mean ± SD)	2.26 ± 1.94
Occurrence of metastases (*n*, %)	
Synchronous	9 (45%)
Metachronous	11 (55%)
Steatosis (%)	45
Inflammation (%)	45
Fibrosis (%)	65

*ASA* American Society of Anesthesiologists, *HVPG* hepatic venous pressure gradient

**Table 2:** Hemodynamic measurements, elastography, laboratory findings, chemotherapy, SOS, and complications

Patient	HVPG	Elastography	Platelets	PT (%)	Chemotherapy (cycles)	OBC	SOS	Complications
1	4.50	6.80	336,000	100	FOLFOX [6]	Yes	Yes	0
2	6.50	N/A	157,000	100	FOLFOX [8]	Yes	Yes	I
3	2.00	N/A	135,000	76	Capecitabine [2]	–	–	0
4	4.00	N/A	105,000	98	FOLFOX [9]	–	Yes	I
5	7.00	7.90	150,000	95	FOLFOXIRI [8]	Yes	Yes	IIIA
6	6.00	8.40	136,000	89	FOLFOX [6]	Yes	Yes	IIIA
7	2.00	N/A	196,000	99	FOLFOX + FOLFIRI [12]	–	–	I
8	2.50	3.80	192,000	100	FOLFOX [4]	Yes	–	I
9	2.50	4.70	199,000	100	Capecitabine [1]	–	–	0
10	1.50	N/A	197,000	100	FOLFOX + bevacizumab [7]	–	–	I
11	5.00	N/A	191,000	100	FOLFOX [4]	Yes	Yes	IIIB
12	2.50	5.20	274,000	100	Capecitabine [6]	–	–	0
13	4.00	4.40	157,000	100	FOLFOX [5]	Yes	Yes	IIB
14	4.00	6.10	135,000	100	FOLFOX [3]	–	–	I
15	4.00	7.60	283,000	100	FOLFOX [6]	Yes	–	I
16	2.50	3.60	215,000	92	FOLFIRI + cetuximab [6]	–	–	I
17	6.00	4.50	291,000	78	TOMOX [6]	Yes	Yes	IIIA
18	2.00	5.20	157,000	100	FOLFOX + TOMOX + panitumumab [8]	–	–	0
19	4.5	4.00	331,000	100	FOLFOX [5]	Yes	Yes	0
20	3	3.50	335,000	62.50	XELOX [5]	Yes	–	IIIB

*N/A* not available

### Results by chemotherapy type

Eight (73%) of the 11 patients receiving preoperative OBC showed SOS lesions in the non-neoplastic hepatic parenchyma. When comparing both groups, patients receiving preoperative OBC had significantly higher values of HVPG and higher SOS development but similar hospital stay, liver stiffness, platelets, and PT. Patients who received preoperative OBC had more major complications than the ones not receiving OBC, with all five patients with major complications receiving OBC (Table 3).

**Table 3:** Comparison of patients with and without oxaliplatin-based chemotherapy

	Chemotherapy without oxaliplatin (*n* = 9)	Oxaliplatin-based chemotherapy (*n* = 11)	*p* value
HVPG (mmHg)^a^ mean (SD)	2.56 (.88)	4.82 (1.44)	0.001
Elastography (kPa)^a^ mean (SD)	4.96 (.91)	5.66 (1.98)	0.478
Platelets (log)^a^ mean (SD)	5.24 (.13)	5.34 (.16)	0.127
PT (%)^b^ median; (IQR)	100.0 (98.0, 100.0)	100 (89.0, 100.0)	0.998
Length of hospital stay (days)^b^ Median (IQR)	7.0 (5.0, 9.0)	8.0 (7.0, 10.0)	0.412
SOS development *n* (%)^c^	1 (11.1)	8 (72.7)	0.010
Major complications *n* (%)^c^	0 (0)	5 (45.5)	0.038

^a^Student’s *t* tests

^b^Mann-Whitney *U*-test

^c^Fisher’s exact test

### Histological findings

Nearly 50% of overall patients in our series presented with histological abnormalities leading to the diagnosis of SOS, all of them having received preoperative OBC. Patients with SOS had higher values of HVPG and had received more chemotherapy cycles, but they had similar liver stiffness, platelets, PT, length of hospital stay, and major complications than those patients without SOS (Table 4).

**Table 4:** Comparison of patients with and without SOS

	No SOS (*n* = 11)	SOS (*n* = 9)	*p* value
HVPG (mmHg)^a^ mean (SD)	2.59 (.80)	5.28 (1.12)	<0.001
Elastography (kPa)^a^ mean (SD)	4.96 (1.40)	6.00 (1.94)	0.266
Platelets (log)^a^ mean (SD)	5.31 (0.13)	5.28 (0.18)	0.698
PT (%)^b^ median (IQR)	100 (92.0, 100)	100 (95.0, 100)	0.882
Length of hospital stay (days)^b^ median (IQR)	7.0 (5.0, 12.0)	8.0 (7.0, 8.0)	0.998
Major complications *n* (%)^c^	1 (9.1)	4 (44.4)	0.127

^a^Student’s *t* tests

^b^Mann-Whitney *U*-test

^c^Fisher’s exact test

### Analysis of complications

Median length of hospital stay was 7 days (range 6–10), and overall complication rate was 70%. The majority of the major complications were basically general (intraabdominal collections, pleural effusions), although two of them were related to biliary leaks that required reoperation However, the majority of patients’ complications (64%) were graded as I–II, and there were no patients with IV or higher grade complications. Four (80%) of the five patients who presented with major complications had SOS on the pathologic analysis. Patients with complications had similar values of HVPG, PT, liver stiffness, platelets, and length of hospital stay than the patients without complications (data not shown). However, patients with major complications had higher values of HVPG and lower PT values but similar liver stiffness, platelets, and length of hospital stay than the patients without major complications (Table 5). Also, major resections were not a factor that was statistically associated with more complications (although nearly significant), age, or comorbidities (defined by the ASA grade). When performing a ROC curve, 4.25 mmHg was the HVPG value that gave better sensitivity and specificity (80 and 80% respectively) for major complications, and the AUC was 0.867 (*p* = 0.016). In this direction, patients with a HVPG of 5 mmHg or greater had more major complications than those under 5 mmHg (20 vs 80%, *p* = 0.005).

**Table 5:** Comparison according to the presence of major complications

	Absent or minor complications (*n* = 15)	Major complications (*n* = 5)	*p* value
HVPG (mmHg)^a^ mean (SD)	3.27 (1.36)	5.40 (1.52)	0.008
Elastography (kPa)^a^ mean (SD)	5.14 (1.33)	6.08 (2.44)	0.511
Platelets (log)^a^ mean (SD)	5.29 (0.15)	5.32 (0.17)	0.772
PT (%)^b^ median (IQR)	100 (99.0. 100.0)	89.0 (78.0, 95)	0.033
Length of hospital stay (days)^b^ median (IQR)	7.0 (6.0, 9.0)	8.5 (7.0, 10.0)	0.445

^a^Student’s *t* tests

^b^Mann-Whitney *U*-test

## Discussion

The appearance of new systemic chemotherapy agents has increased resectability and survival rates in patients with CLM, but some of these agents damage the liver parenchyma and increase postoperative morbidity and mortality [4, 5, 15]. One of the most well-known liver injury patterns is oxaliplatin-induced SOS, which can evolve into non-cirrhotic PHT in serious cases [6]. However, no studies have shown the hemodynamic effects of chemotherapy-induced hepatic toxicity by direct measurement of the vascular pressures by transjugular hemodynamic procedure of the liver, or quantified the association between these hemodynamic effects and postoperative complications [7, 8].

Oxaliplatin is a systemic chemotherapy agent that prevents cell division and replication by crosslinking DNA strands. In the liver, oxaliplatin damages mainly the sinusoidal endothelial cells and causes SOS, which ultimately decreases the hepatic functional reserve and increases the risk of bleeding during surgery [4, 5]. Several studies have intended to predict SOS based on blood and serum parameters like platelet count, aspartate aminotransferase, gamma glutamyl transferase, alkaline phosphatase, and aspartate aminotransferase to platelet ratio index. However, these biomarkers have not been proved useful to quantify the degree and extent of hepatic injury, and still no large studies have validated them as a reliable indicator of sinusoidal damage [7, 8]. On the other hand, there are no studies focused on the presence of clinical or subclinical PHT induced by OBC, something that only direct measurement of liver hemodynamic parameters can accurately diagnose [11]. Our study showed that patients receiving preoperative OBC had higher values of HVPG when compared to patients not receiving OBC and also that SOS development was associated with receiving OBC (especially when the number of chemotherapy cycles increased). This latter observation is consistent with previous studies showing a clear link between OBC and the development of SOS [4, 6, 16, 17]. On the other hand, the increase of HVPG in patients receiving OBC or developing SOS clearly shows that OBC impairs liver pathology and hemodynamic parameters. This observation favors the logical and already postulated pathogenical hypothesis that preoperative OBC would lead to sinusoidal obstruction and increased intrahepatic resistance and would subsequently increase HVPG [6, 17].

Several studies have highlighted the increase of postoperative morbidity and mortality in CLM patients treated with chemotherapy drugs and the important role of a proper operative timing given the frequent attenuation of liver injury after stopping the systemic chemotherapy agent [18–20]. The ideal situation would be to predict the increased postoperative risk before surgery by diagnosing SOS in patients treated with OBC. To this objective, some authors have proposed to perform preoperative percutaneous or transjugular liver biopsies or even a diagnostic laparoscopy to rule out significant liver alterations and select the best possible therapeutic strategy for each patient [5, 18, 19]. However, as SOS histological lesions may be focal or even patchy, these alternatives remain controversial because of the risks of sampling error and subsequent underdiagnosis of sinusoidal lesions. Lately, Nakano et al. have identified several preoperative factors associated to sinusoidal damage (6 or more cycles of oxaliplatin, abnormal preoperative liver function tests, increased indocyanine green retention rate, and female sex) that can help to get the diagnosis in the preoperative setting [20]. In our study, however, we could not find differences in the preoperative biochemical parameters between patients developing SOS or not, nor did we find differences in the major complication rate between patients developing SOS. Nevertheless, the specific aim of our study and the limited number of patients included in it can easily explain these observations because we designed our study towards successfully finding differences in hemodynamic alterations but not towards finding differences in the preoperative biochemical parameters or in major complications. Despite this limitation, that adds to not being able to prove that increases in HVPG are the main cause for postoperative complications, we believe that the large amount of existing studies proving the relation between SOS development and the subsequent alteration of preoperative biochemical parameters (with increased complications) makes it unnecessary to challenge this well-studied fact and still keeps the validity of our study.

PHT becomes clinically significant when HVPG rises above 10 mmHg and the patient develops cirrhotic complications like variceal bleeding, ascites, or thrombocytopenia [11]. Moreover, several studies on patients with liver cirrhosis undergoing liver resection and other surgical procedures have demonstrated the importance of PHT. In this direction, more than 50% of cirrhotic patients classified as Child-Pugh A will decompensate after surgery, with subsequent decreased quality of life and long-term survival [21–23]. Although splenomegaly and thrombocytopenia are usually indirect clinical signs of PHT in cirrhosis or in patients receiving OBC [6–8], direct hepatic hemodynamic evaluation is currently the best method to study and diagnose PHT [11, 24]. To the present one, no study had directly measured the liver hemodynamic effects of chemotherapy-induced liver damage or attempted to correlate their hemodynamic changes with the development of complications. In our study, patients with major complications had higher HVPG than those not developing major complications, and major complications developed at lower cutoff HVPG levels than the usually accepted to define clinically significant PHT in patients with chronic liver diseases [11, 24]. These interesting new findings suggest a causal association between OBC-induced liver hemodynamic impairment and major complications but also show that the development of major complications in CLM patients receiving preoperative OBC starts at earlier HVPG levels than those universally accepted as clinically relevant in patients with chronic liver diseases. Although the design of our study cannot elucidate the cause for this earlier manifestation of initial hemodynamic impairment, we hypothesize that OBC-related liver injury would cause significant liver damage and an increase in major postoperative complications because of the lack of compensatory mechanisms against the hemodynamic disturbances that only develop in patients with chronic liver diseases. More important, however, it is the fact that clinicians taking care of CLM patients receiving OBC should be aware of the development of this early significant hemodynamic impairment and specifically look for it in advance because of its severe clinical consequences.

Elastography has been advocated to be a useful tool to diagnose the degree of liver fibrosis and PHT through liver stiffness measurement, although several factors including sex, liver inflammation, cholestasis, hepatocellular carcinoma, liver congestion, and disease etiology may influence its results [12, 25–27]. One of the few studies about liver elastography in patients with CRC has shown that liver stiffness increases within 48 h after starting OBC and normalizes within 2 weeks after treatment in most cases [28]. In our study, we could not find any difference in liver stiffness when comparing by whether the patient had received OBC, developed SOS or suffered major complications. The most likely reason for this lack of association is that, as observed in other studies, elastography results only correlate with those obtained by hepatic hemodynamic evaluation for HVPG above 10 mmHg [12, 25–27]. Also, elastography results are mainly affected by the amount of liver fibrosis but not by the degree of sinusoidal obstruction, a fact that is better studied with direct liver hemodynamic measurements [11, 29]. We believe that in order to find some differences in liver stiffness, our study should have included patients with higher hemodynamic impairment, something that was not done because of the exploratory nature of our study. However, this limitation needs to be taken into account when designing further studies specifically aiming to find the value of elastography to predict chemotherapy-induced liver injury.

Even though this is the first study to directly analyze liver hemodynamic parameters in CLM patients receiving preoperative chemotherapy, we acknowledge that our study had some limitations. Small sample size is probably the most obvious and important one, but this limitation comes from the study being necessarily an exploratory analysis because no similar studies existed and because potential complications that can derive from direct hepatic hemodynamic measurement advised against a large preliminary study [29]. Nevertheless, we feel that this is a minor limitation because sample size accounted for not finding differences in some variables that have extensively described by other studies but that were not the primary objective of our study. In this sense, future similar studies should aim to enroll more patients and include more heterogeneous patients (e.g., patients with previous chronic liver diseases or with longer chemotherapy treatments) in order to enable stratification and ascertain the effect of other preoperative characteristics in the postoperative results.

Other limitations that can be found in our study include the concern for overdiagnosis of SOS after OBC, which derives from the fact that nearly 75% of patients receiving OBC were histologically diagnosed with SOS, and also the fact that HVPG measurement was done only after receiving chemotherapy, raising the concern that maybe there were some prior alterations in HVPG before chemotherapy. We feel that further studies with different designs adapted to these limitations can help to solve these questions that our current design study cannot answer.

## Conclusions

To conclude, our preliminary study confirmed the association between OBC and SOS in CLM patients and showed that OBC-induced SOS impairs liver hemodynamics, moreover describing that major complications in these patients start to develop at lower HVPG levels than those universally accepted as clinically relevant in patients with chronic liver diseases. These interesting results should encourage further analyses to define the effect of other preoperative characteristics in CLM patients receiving OBC and thus improve postoperative results.

## Abbreviations

CLM: Colorectal liver metastases
CRC: Colorectal carcinoma
HVPG: Hepatic venous pressure gradient
OBC: Oxaliplatin-based chemotherapy
PHT: Portal hypertension
SOS: Sinusoidal obstructive syndrome
